# Supplementary material for: An Integrated Approach for the Early Detection of Endometrial and Ovarian Cancers (Screenwide Study): Rationale, Study Design and Pilot Study
Source: J Pers Med. 2022 Jun 29;12(7):1074. doi: 10.3390/jpm12071074 (PMC9324683; doi:10.3390/jpm12071074)
Supplement: Supplementary file 1 [file jpm-12-01074-s001.zip › jpm-1771748-File S1.pdf]

SUPPLEMENTAL MATERIAL

Panel design

We constructed a panel of exonic regions of 49 genes in order to perform a pilot study. The panel estimated coverage was 247 kb. We finally excluded two genes (MUC16 and AHNAK2), because of they were no clear drivers for EC or OC and accumulated several false positive calls. We constructed the libraries using duplex unique molecular identifiers (UMIs) (Integrated DNA Technologies, Inc-IDT, Coralville, IA, USA), an amplicon-based target enrichment method designed to detect low-frequency allelic variants by the addition of a degenerate 3-nucleotide-long molecular barcode index to the captured DNA fragments, given that we expected to find a very little amount of tumour cells in these samples [1].

DNA isolation: DNA samples from all types of specimens were isolated as follows. Approximately 5 ml and 1.5 ml of cervical Pap-brush samples and vaginal self-samples, respectively, were centrifuged for 20 minutes at 11.000 rpm. Also, 300 µl of the buffy coat obtained from 10 ml of blood was pre-incubated at room temperature for 5 min at 180 rpm. Prior to DNA extraction, the resulting centrifuged cell pellet and the pre-incubated buffy coat were digested with proteinase K, provided by the extraction kit (Maxwell 16 Lev Blood kit, Promega Corporation, Madison, WI, USA), at 56 ºC for 20 min. DNA from cervical Pap-brush samples and vaginal self-sampling was extracted and finally eluted in 50 µl of nuclease free water. Likewise, DNA from buffy coat was extracted and eluted in 300 µl of nuclease free water. Last, approximately 50 mg of frozen aspirate biopsies and surgical resection specimens (tumours) was used to extract DNA using Maxwell 16 Tissue DNA Purification kit (Promega Corporation, Madison, WI, USA) and eluted in 300 µl of nuclease free water. All DNA samples were isolated using the automated nucleic acid purification system Maxwell® 16 Instrument (Promega Corporation, Madison, WI, USA) that processes up to 16 samples. DNA concentration was determined using the Qubit dsDNA Broad Range Assay Kit (Thermo Fisher Scientific, Waltham, MA, US).

Preparation of the libraries and sequencing

The range of input DNA to prepare de libraries spanned from 150 ng to 1 μg. In cases where DNA concentrations were lower than 4.75 ng/μl, a 3-times ratio of AMPure XP Bead reagent (Beckman Coulter, Brea, CA, USA) was used to concentrate the samples as manufacter's instructions indicate. Input DNAs were enzymatically fragmented using the Frag Enzyme from KAPA HyperPlus Library Preparation Kit (Roche Sequencing Solutions, Inc., Pleasanton, CA, USA). Incubation times ranged from 20 to 25 min at 37ºC in order to achieve 150-350 bp fragments. After enzymatic fragmentation, end-repair and A-tailing (KAPA HyperPlus Library Preparation kit, (Roche Sequencing Solutions, Inc., Pleasanton, CA, USA)) were performed to DNA fragments before ligation of barcoded adaptors. The resulting A-tailed fragments were ligated to the xGen CS Adapters (IDT, Coralville, IA, USA). Duplex Seq adapters contain an equimolar pool of 64 pairs of duplexed adapters, each containing a 3 bp unique molecular identifier (UMI). For each library, 75 pmol of adapters was added and ligated for 15 min at 20 ºC. Before amplification, libraries were cleaned-up and double-sided size selection was performed with AMPure XP bead reagent (Beckman Coulter, Brea, CA, USA) (first side at 0.7X and second side at 0.9X). Next, libraries were PCR amplified using a final concentration of 4 μM of UDI Dup Seq amplification primers (IDT) and performing 8 cycles of PCR (except for 4 samples where the repetition was performed 7 times). Upon a clean-up step with AMPure XP bead reagent (Beckman Coulter, Brea, CA, USA), the eluted libraries were quantified by using a Qubit fluorometer (Invitrogen, Life Technologies, Carlsbad, CA, USA) and the quality of DNA was measured with the Agilent Bioanalyzer DNA 1000 assay (Agilent, Santa Clara, CA, USA) at the Unitat de Genòmica, Centres Científics i Tecnològics de la Universitat de Barcelona (CCiTUB).

Prior capture, libraries were multiplexed in groups of 7, 10, 12 or 13 samples, reaching a final amount of DNA between 1.5 and 2.16 μg. That means a total amount of each library of 125, 166, 200 or 285 ng, depending on the total amount of libraries multiplexed. DNA blocking of the

multiplexed libraries was performed using 5 μg of COT Human DNA provided by the HyperCap Enrichment Kit (Roche) and 2 μl of xGen Universal Blockers TS-Mix (IDT, Coralville, IA, USA). Multiplexed libraries were then hybridised to the SeqCap EZ probe pool (NimbleGen, Roche) for 16-20 h at 47 ºC. Hybridization samples were then captured using the KAPA HyperCap Enrichment Kit (Roche Sequencing Solutions, Inc., Pleasanton, CA, USA) and the HyperCap Bead kit (Roche Sequencing Solutions, Inc., Pleasanton, CA, USA) following manufacturer's protocol. Captured DNA was PCR amplified using xGen Library Amplification Primer Mix (IDT, Coralville, IA, USA) for a total of 14 cycles. Amplified captures were purified with AMPure XP Bead reagent (Beckman Coulter) and eluted with 50 μl of 10 mM Tris-HCl pH 8.0. Finally, the captured pools were quantified using a Qubit fluorometer (Invitrogen, Life Technologies, Carlsbad, CA, USA) and the quality of DNA was measured with the Agilent Bioanalyzer DNA 1000 assay (Agilent, Santa Clara, CA, USA).

Deep sequencing was performed at high depth (10.000x, approximately) on two different platforms with different conditions: (i) on an Illumina HiSeq3500 sequencer (Illumina, San Diego, CA, USA) using the 100 bp paired-end sequencing protocol, and (ii) on a Illumina Novaseq (Illumina, San Diego, CA, USA) using SP flow cell and 150 bp paired-end sequencing protocol at the Centre Nacional d'Anàlisi Genòmica (CNAG).

Processing sequence data with UMIs

Raw FASTQ files for each read pair were obtained with UMI sequence in line and were processed using IDT guidelines. FASTQ files were converted to unaligned BAM files and Fgbio's [2] ExtractUmisFromBam command then was used to process unmapped BAM files and extract UMI sequences. A combination of bwa mem aligner [3] and Picard's MergeBamAlignment tool [4] was applied to generate a mapped BAM that included all necessary metadata. Reads were grouped with fgbio GroupReadsByUmi. This tool identifies which reads come from the same source molecule by combining UMI sequence and mapping position.

Unmapped consensus reads were generated from the output of GroupReadsByUmi and using fgbio's CallDuplexConsensusReads, for duplex strategy. Reads with the same unique molecular tag were first assembled into single strand consensus molecules. For molecules that have at least one observation in each strand, duplex consensus reads were generated by combining the evidence from the two single strand consensus reads. Duplex consensus reads were required to be constructed from at least 2 reads, 1 read for each single-strand. After generating consensus reads, reads were remapped using the same mapping procedure as for raw reads described above and filtered out by minimum mean base quality [5] and maximum consensus error rate (0.05 per read and 0.1 per base) generated in the consensus formation process. Overlap between reads was eliminated to ensure that in downstream processes, particularly variant calling, overlapped bases in the paired end reads are not double counted.

Variant calling and filtering

Two variant callers were used to increase the precision of the detection of variants and to reduce false positive callings. Single nucleotide variants (SNV) and small insertions and deletions (indels) were called using VarDictJava [6] and Mutect2 [7]. Variant callers were used with default parameters and the following slight modifications: we prevented VartDictJava to perform local realignment and Mutect2 was forced to not consider soft-clipped-bases. Variants were filtered by quality parameters (mean mapping quality above 50, mean position in the read above 9 and the maximum number of events in the haplotype to 7). We set the minimum variant frequency to 0.005, the minimum read depth to make a call to 100 and minimum number of reads supporting the variant to 4. Moreover, a list of recurrent artefacts (found in at least 2 samples from controls or blood samples from EC patients) was generated and used to filter out false positive variants. Aligned reads for each variant was reviewed manually in IGV [8] to confirm the variant call.

Variants were gene-based and filter-based annotated using ANNOVAR (RefSeq Gene, ExAC, 1000 Genomes Project, gnomAD, CLINVAR, InterVar, dbSNP, REVEL, dbNSFP,

COSMIC)[9,10]. Variants found in population frequency above 0.1% in annotated databases (gnomAD and ExAC, continental populations except Finish, Ashkenazy and those described as "other") were filtered out as these variants are probably polymorphic. Since blood DNA would not be routinely collected for larger cohorts of EC patients (tumour-only strategy), this can be used as a strategy to filter out putative germline variants in tumours and aspirate biopsies. However, in this pilot study variants detected in blood samples with VAF >30% were considered germline variants and filtered out in all samples. Since in uterine aspirates somatic variants that are clonal in the tumour are also found at very high frequency whereas low-frequency variants in this type of sample can be representative of clones carrying driver-mutations in normal tissue [11], a specific sample type filter were applied in aspirates to enrich for somatic mutations originated in tumours and only variants with VAF above 5% were included. To better interpret the variation found in the samples, mutations were analysed with Cancer Genome Interpreter (CGI) [12], a platform designed to identify tumour alterations that drive the disease. According to CGI results, variants identified were classified as known drivers of EC, predicted drivers or passengers. We evaluated the performance and sensitivity of variant calling using variants identified in the tutor sample with VAF > 5% as our set of truth variants. This set of variants was then screened in paired aspirates and minimally invasive samples to assess the sensitivity of our approach.

References

1. Costas, L.; Frias-Gomez, J.; Guardiola, M.; Benavente, Y.; Pineda, M.; Pavón, M.Á.; Martínez, J.M.; Climent, M.; Barahona, M.; Canet, J.; et al. New Perspectives on Screening and Early Detection of Endometrial Cancer. *Int. J. Cancer* **2019**, *145*, 3194–3206. https://doi.org/10.1002/ijc.32514.
2. Fulcrum Genomics Fgbio; 2020. Available online: http://fulcrumgenomics.github.io/fgbio/. Accessed on 29 May 2021.
3. Li, H. Aligning Sequence Reads, Clone Sequences and Assembly Contigs with BWA-MEM. *arXiv* **2013**, arXiv:1303.3997.
4. Broad Institute Picard Toolkit; Broad Institute, GitHub repository, 2019. Available online: https://broadinstitute.github.io/picard/. Accessed on 28 May 2021.
5. Smith, T.; Heger, A.; Sudbery, I. UMI-Tools: Modeling Sequencing Errors in Unique Molecular Identifiers to Improve Quantification Accuracy. *Genome Res.* **2017**, 27, 491–499. https://doi.org/10.1101/gr.209601.116.

6. Lai, Z.; Markovets, A.; Ahdesmaki, M.; Chapman, B.; Hofmann, O.; McEwen, R.; Johnson, J.; Dougherty, B.; Barrett, J.C.; Dry, J.R. VarDict: A Novel and Versatile Variant Caller for next-Generation Sequencing in Cancer Research. Nucleic Acids Res. **2016**, 44, e108–e108. https://doi.org/10.1093/nar/gkw227.
7. Benjamin, D.; Sato, T.; Cibulskis, K.; Getz, G.; Stewart, C.; Lichtenstein, L. Calling Somatic SNVs and Indels with Mutect2; *bioRxiv*, 2019, 861054. https://doi.org/10.1101/861054. Available online: https://www.biorxiv.org/content/10.1101/861054v1. Last accessed on 22 June 2022.
8. Robinson, J.T.; Thorvaldsdóttir, H.; Wenger, A.M.; Zehir, A.; Mesirov, J.P. Variant review with the Integrative Genomics Viewer. *Cancer Res*. **2017**, 77, e31–e34. https://doi.org/10.1158/0008-5472.CAN-17-0337.
9. Wang, K.; Li, M.; Hakonarson, H. ANNOVAR: Functional Annotation of Genetic Variants from High-Throughput Sequencing Data. *Nucleic Acids Res*. **2010**, 38, e164. https://doi.org/10.1093/nar/gkq603.
10. Yang, H.; Wang, K. Genomic Variant Annotation and Prioritization with ANNOVAR and WANNOVAR. *Nat. Protoc*. **2015**, 10, 1556–1566. https://doi.org/10.1038/nprot.2015.105.
11. Moore, L.; Leongamornlert, D.; Coorens, T.H.H.; Sanders, M.A.; Ellis, P.; Dentro, S.C.; Dawson, K.J.; Butler, T.; Rahbari, R.; Mitchell, T.J.; et al. The Mutational Landscape of Normal Human Endometrial Epithelium. *Nature* **2020**, 580, 640–646. https://doi.org/10.1038/s41586-020-2214-z.
12. Tamborero, D.; Rubio-Perez, C.; Deu-Pons, J.; Schroeder, M.P.; Vivancos, A.; Rovira, A.; Tusquets, I.; Albanell, J.; Rodon, J.; Tabernero, J.; et al. Cancer Genome Interpreter Annotates the Biological and Clinical Relevance of Tumor Alterations. *Genome Med*. **2018**, 10, 25. https://doi.org/10.1186/s13073-018-0531-8.
